# Supplementary material for: Identification of photoperiod-induced specific miRNAs in the adrenal glands of Sunite sheep (Ovis aries)
Source: Front Vet Sci. 2022 Jul 22;9:888207. doi: 10.3389/fvets.2022.888207 (PMC9354845; doi:10.3389/fvets.2022.888207)
Supplement: Supplementary file 1 [file Data_Sheet_1.ZIP › Supplementary file/miRDeep2 file.zip!/miRDeep2 file/Know_Structure/SPLP42A1/oar-mir-495.pdf]

miRBase precursor : oar-mir-495
Total read count : 15607
oar-miR-495-5p read count : 98
oar-miR-495-3p read count : 15487
remaining reads : 22

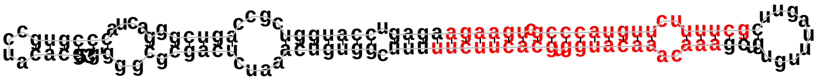

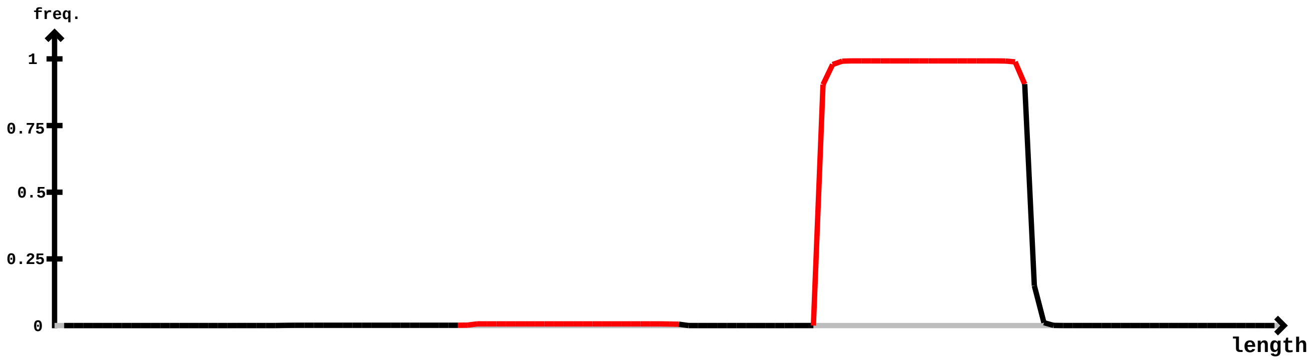


```
                                                                                            oar-miR-495-3p
                                          oar-miR-495-5p
5'- gcacauccgugcccaucagggcugaccgcugguaccugagaagaagucgcccauguucuuuucgcuugauuugugucgaaacaaacauggugcacuucuuuuucggugucaaaucucagcgcgggg   -3'   exp
    ((((....))))((....(.(((((....(((((((.((((((((((.(((((((((..((((((.........).)))))..))))))).))))))))))))).)))))))....))))).)..))   reads  mm
    .................agggcugaccgcugguaccugagaa....................................................................................   1      0
    ......................ugaccgcugguaccugaga.....................................................................................   1      0
    ......................ugaccgcugguaccugagaa....................................................................................   11     0
    .......................gaccgcugguaccugagaa....................................................................................   4      0
    ........................accgcugguaccugagaa....................................................................................   1      0
    .........................................agaagucgcccauguucuuuu................................................................   4      0
    .........................................agaagucgcccauguucuuuuc...............................................................   2      0
    .........................................agaagucgcccauguucuuuucg..............................................................   3      0
    ..........................................gaagucgcccauguucuuuu................................................................   2      0
    ..........................................gaagucgcccauguCcuuuu................................................................   1      1
    ..........................................gaagucgcccauguucuuuuc...............................................................   10     0
    ..........................................gaagucgcccauguucuuuucg..............................................................   75     0
    ..........................................gaagucgcccauguucuuuucgc.............................................................   1      0
    ...........................................................................ucgaaacaaacauggugcacuu.............................   1      0
    ...........................................................................ucgaaacaaacauggugcacuucuu..........................   1      0
    ..............................................................................aaacaaacauggugcacuA.............................   1      1
    ..............................................................................aaacaaacauggugcacuu.............................   5      0
    ..............................................................................aaacaaacauggugcacuuc............................   47     0
    ..............................................................................aaacaaacauggugcacuucu...........................   1220   0
    ..............................................................................aaacaaacauggugcacuucuu..........................   11264  0
    ..............................................................................aaacaaacauggugcacuucuuu.........................   1528   0
    ..............................................................................aaacaaacauggugcacuucuuuu........................   35     0
    ...............................................................................aacaaacauggugcCcu..............................   1      1
    ...............................................................................aacaaacauggugcacuu.............................   1      0
    ...............................................................................aacaaacauggugcacuuc............................   3      0
    ...............................................................................aacaaacauggugcacuucu...........................   79     0
    ...............................................................................aacaaacauggugcacuucuu..........................   501    0
    ...............................................................................aacaaacauggugcacuucuuu.........................   573    0
    ...............................................................................aacaaacauggugcacuucuuuu........................   17     0
    ...............................................................................aacaaacauggugcacuucuuuuu.......................   1      0
    ................................................................................acaaacauggugcacuucu...........................   3      0
    ................................................................................acaaacauggugcacuucuu..........................   31     0
    ................................................................................acaaacauggugcacuucuuu.........................   62     0
    ................................................................................acaaacauggugcacuucuuuu........................   99     0
```

```
                                                                                 oar-miR-495-3p
                                         oar-miR-495-5p
gcacauccgugcccaucagggcugaccgcugguaccugagaagaagucgcccauguucuuuucgcuugauuugugucgaaacaaacauggugcacuucuuuuucggugucaaaucucagcgcgggg

................................................................................acaaacauggugcacuucuuuuu.......................    2    0
.................................................................................caaacauggugcacuucuuu.........................    1    0
.................................................................................caaacauggugcacuucuuuu........................    7    0
.................................................................................caaacauggugcacuucuuuuu.......................    6    0
....................................................................................................uuucggugucaaaucucagcg.....    1    0
....................................................................................................uuucggugucaaaucucagcgcgg..    1    0
```
